# Prescription Design of Sinomenine Gel Based on Molecular Dynamics Simulations

**DOI:** 10.3390/ijms252312863

**Published:** 2024-11-29

**Authors:** Yiran Kang, Wei Shen, Shili Pan, Haiying Lian, Xuehui Ding, Jingying Li, Jiaoyue Zhu, Lin Wang, Wei Xu

**Affiliations:** School of Pharmacy, Changchun University of Chinese Medicine, Changchun 130117, China; a19843528798@163.com (Y.K.); 15124342946@163.com (W.S.); 15704470610@163.com (S.P.); 19291886106@163.com (H.L.); 15837573625@163.com (X.D.); 18043910903@163.com (J.L.); 15834878190@163.com (J.Z.)

**Keywords:** molecular dynamics simulations, prescription screening, sinomenine gel, molecular mechanism

## Abstract

Sinomenine (SIN) is a drug for the treatment of rheumatoid arthritis, most of which is administered orally, but it is prone to adverse gastrointestinal effects. Gel can overcome the gastrointestinal adverse effects caused by oral administration. In this paper, a multiscale computational pharmaceutics strategy was developed to guide the systematic study of formulation factors of a SIN gel and, further, to guide the formulation design. A molecular dynamics (MD) simulations method was utilized to successfully screen the optimal prescription of SIN gel and to elucidate the molecular mechanisms affecting the quality of SIN gel. The optimal prescription was 3.0% of SIN, 1.0% of Carbopol (Cp), 30% of Ethanol (Eth), 5.0% of Glycerine (Gly) and 10.0% of Menthol (Men). The influence mechanism can be explained by the combination of multiple parameters, such as the microstructure diagram, the radius of gyration (Rg) and the radial distribution function (RDF). In vitro transdermal studies were carried out using a modified Franz diffusion cell method to evaluate the quality of the screened and reference prescriptions. The results showed that the cumulative penetration and penetration rate of the screening of prescription were better than the reference formulation. Most important of all, the simulation results are in good agreement with the in vitro release experiment, indicating that the strategy has good applicability. This study was able to accurately optimize the formulation and elucidate the molecular mechanism, which would provide a reference for further research on SIN and gel.

## 1. Introduction

Sinomenine (SIN), a poorly soluble alkaloid isolated from traditional Chinese medicine Caulis sinomenii, possesses good analgesic, anti-inflammatory and immunomodulatory effects and is non-addictive [1]. Presently, SIN is mainly used as an oral preparation for the treatment of diseases, which can cause gastrointestinal discomfort when taken for a long period of time. Gel is a transdermal drug delivery agent, which can work locally and reduce systemic adverse effects, and the preparation process is simple [2,3]. Transdermal means the application of a medicine or drug through the skin. In the simplest terms, a drug is placed on top of the skin, where it is absorbed into the bloodstream. It has been found that the preparation of SIN gel has better therapeutic effects [4,5]. Currently, the development of transdermal formulations of SIN is still in the experimental research stage. As there is no officially marketed transdermal delivery product of SIN, preparing SIN gel as a transdermal formulation in this study holds practical significance [6,7]. Throughout the past few decades, notable strides have been made in the development of drug formulations within specific research fields. However, current methods are still based on traditional trial-and-error approaches. These methods are characterized by long time periods, the need for large investments and results that are fraught with uncertainty [8]. In recent years, molecular dynamics (MD) simulations have become very popular in the development of drug formulations. As the means closest to laboratory conditions in molecular level simulation, MD simulations can accurately characterize the micro dynamic changes of a system at the atomic level and vividly reveal the mechanisms and laws behind macroscopic phenomena in experiments, driving research toward greater efficiency, economy and predictability [9,10]. The application of MD in formulation prescription development is an effective complement to traditional experimental methods. The two validate each other, enabling formulation development to support and improve each other in macroscopic and microscopic studies [11,12]. In this study, SIN was taken as the research foundation. Based on MD simulations, SIN gel was designed at the molecular level. The optimal prescription of SIN gel was predicted, the principles of molecular mechanisms influencing the quality of SIN gel were elucidated, and, finally, the transdermal properties were evaluated. This study rapidly and accurately develops SIN gel dosage with good patient compliance and provides new research ideas for other scientific studies in the field of formulation.

## 2. Results

### 2.1. Solubility Parameter (δ)

The solubility parameter (δ) was measured in the experiment or calculated using experimental correlations or MD methods. And δ can be viewed as a great benchmark for selection of the solvent with best performance. In this study, δ values were calculated for Carbopol (Cp), chitosan (CHI) and carboxymethylcellulose (CMC), which are commonly used as matrices for gel. The smaller the difference in δ, the more compatible. As can be seen in Table 1, the order of compatibility between the matrix and SIN is Cp > ChI > CMC. Although Cp is more compatible in comparison, ∆δ is still greater than 7.5 (J/cm^3^)^1/2^, probably due to the effect of degree of aggregations. Therefore, the δ of Cp at n = 2, 10, 20, 30, 40 and 50 was calculated using the “Equal solubility parameter substitution method”, and the trend of the change is shown in Table 2, and the δ no longer changed when the degree of aggregations were increased to 30, which indicated that the Cp could be used to substitute for the polymer at n = 30. At this time, δ = 23.622 ((J/cm^3^)^1/2^, ∆δ = 3.665 < 7.5 (J/cm^3^)^1/2^, so it is considered that SIN and Cp have better compatibility.

### 2.2. Influence of Cp Degree of Aggregations on the Quality of SIN Gel

**Figure 1:**
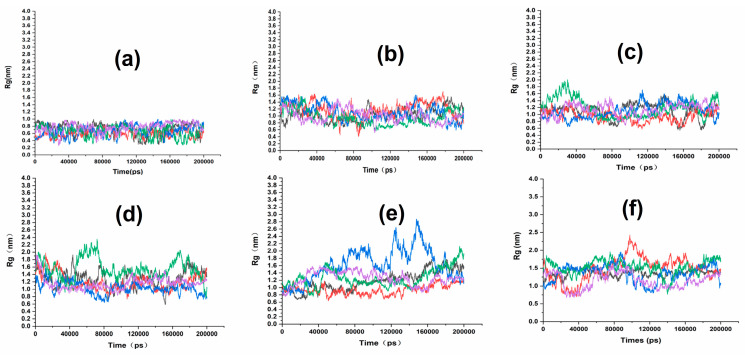
Rg variation curves for Cp with different degree of aggregations. (**a**) n = 15; (**b**) n = 30; (**c**) n = 40; (**d**) n = 50; (**e**) n = 60; (**f**) n = 70.

**Figure 2:**
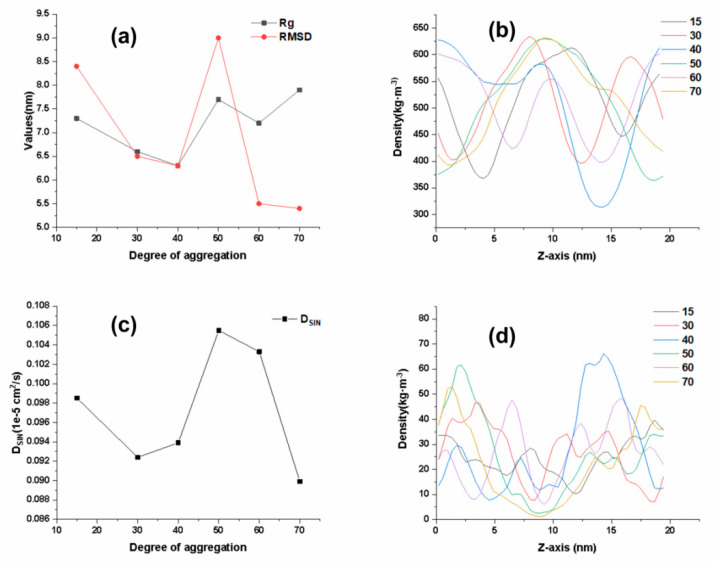
Effect of Cp with different degree of aggregations on SIN gel. (**a**) Rg and RMSD variation curves for Cp with different degree of aggregations; (**b**) density distribution in the z-axis direction for Cp with different degree of aggregations; (**c**) diffusion coefficient for Cp with different degree of aggregations; (**d**) density distribution of solvent water in the z-axis direction for Cp with different degree of aggregations.

Based on the results of the previous study, Cp was identified as the best matrix. The present study was designed to investigate the effect of different degree of aggregations on gel at 15, 30, 40, 50, 60 and 70 [13]. As shown in Figure 1, the smaller the polymerization of Cp, the smaller the radius of gyration (Rg) value of the matrix chain. This indicates that the degree of swelling of the matrix chain in water, which describes the crosslinked mesh structure of the gel, is not consistent. Therefore, when determining the degree of aggregations, a polymer matrix chain with a larger degree of aggregations should be selected for study. Analysis of the results of the changes in diffusion coefficient with polymerization reveals (Figure 2c) that between 40 and 60 is beneficial for the diffusion of SIN drug molecules in the SIN gel matrix. This trend is consistent with the results of the changes in the Rg and root mean square deviation (RMSD) of polymer matrix chains of different degrees of aggregations with the polymerization, as shown in Figure 2a. The larger the Rg and the RMSD indicates that the polymer swells more in the gel system, which indicates that it forms gel voids and that its swelling is not consistent with its crosslinking. It indicates that the larger the gel void it forms is more favorable for solvent storage, and, the more solvent is present, the faster the drug diffuses. Further analysis of the density distribution of the polymer matrix chain with respect to the degree of aggregations and the density distribution of the solvent water with respect to the polymerization degree, as shown in Figure 2b,d, reveals that the polymer matrix chain with a polymerization degree of 50 forms the largest area in terms of density distribution. Thus, the polymer matrix chain formed has the largest voids, with a better water storage effect. Drug molecules in the system are more likely to be released from the gel polymer matrix network. In conclusion, the optimum degree of aggregations for the Cp polymer is determined to be 50.

### 2.3. Effect of SIN Concentration on the Quality of SIN Gel

**Figure 3:**
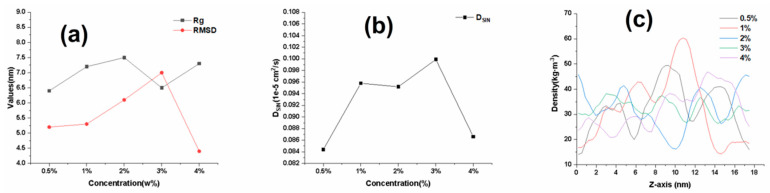
Effect of different SIN concentrations on the quality of SIN gel. (**a**) Rg and RMSD variation curves for different SIN concentrations; (**b**) diffusion coefficient for different SIN concentrations; (**c**) density profiles in the z-axis direction for different SIN concentrations.

In order to determine the optimal amount of SIN as well as to elucidate the molecular mechanism, simulation systems with SIN concentrations of 0.5%, 1%, 2%, 3% and 4% were designed. As shown in Figure 3a, the Rg and RMSD of the matrix chain gradually increased when the SIN concentration was increased from 0.5% to 3%, and the trends of Rg and RMSD changed significantly when the concentration exceeded 3%. The fluctuation of the change in RMSD became smaller, indicating that the system reached an equilibrium state. Analysis of the Rg of the matrix chain showed that the Rg of the matrix chain is gradually increasing as the concentration of SIN increases. This indicates that the matrix chain undergoes swelling and the structure of the gel network formed is more conducive to the presence of water in the gel matrix network, and drug molecules are more easily released. As shown in Figure 3b, when the concentration of SIN is increased to 4%, the diffusion coefficient of SIN in the system drops significantly. At this time, the optimum concentration of SIN cannot be determined. As shown in Figure 3c, it is clear that at SIN concentrations of 3% and 4%, the density profile of the matrix chain is significantly more uniform compared to other SIN concentrations. In a state where the gel uniformity is consistent, an increase in SIN concentration causes SIN molecules to displace water molecules within the gel matrix network. As a result, the reduction in water molecules within the gel matrix chain network impedes the release of SIN, leading to a significant decrease in the diffusion coefficient of SIN at these concentrations. In summary, the optimal concentration of SIN is 3%.

### 2.4. Effect of Cp Concentration on the Quality of SIN Gel

**Figure 4:**
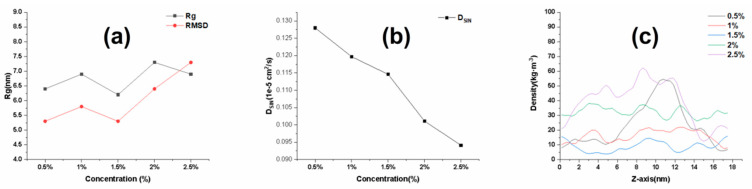
Effect of different polymer matrix concentrations on the quality of SIN gel. (**a**) Rg and RMSD curves variation for different Cp concentrations; (**b**) diffusion coefficient for different Cp concentrations; (**c**) density profiles in the z-axis direction for different Cp concentrations.

As shown in Figure 4a, the Rg and RMSD of the matrix chain are increasing with the increase in matrix concentration. This is because the matrix chain is a long-chain molecule. When the concentration is increased, the molecular activity space becomes smaller, and the matrix chains become entangled with each other. This results in slower movement of the matrix chain in the system and makes the system become homogeneous. This also verifies the phenomenon that the gel becomes more viscous as the matrix concentration increases during the preparation of gel. Analyzing the diffusion coefficient of drug molecules in the system with different matrix concentrations, the diffusion coefficient decreases gradually with the increase in matrix concentration, as shown in Figure 4b. The polymer matrix concentration influences the homogeneity of the density distribution in the z-axis direction of the SIN gel system. From Figure 4c, it can be seen that the distribution of the gel system is more homogeneous when the concentration of the matrix is between 1% and 2%. Combined with the pre-test as well as the simulation results, the optimal concentration was determined to be 1%. The Rg values of the matrix chains in the systems with different matrix concentrations versus time curves are shown in Figure S1.

### 2.5. Effect of Ethanol Concentration on the Quality of SIN Gel

**Figure 5:**
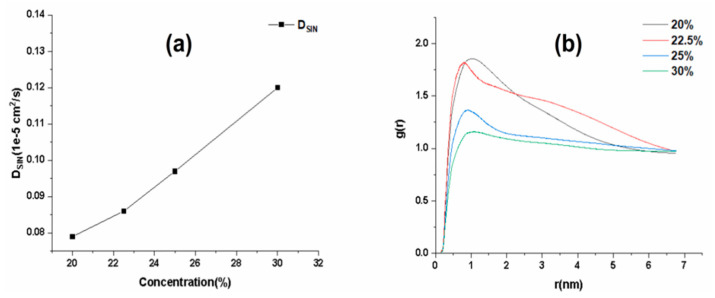
Effect of different Eth concentrations on the quality of SIN gel. (**a**) Diffusion coefficient for Eth concentrations; (**b**) RDF values for different Eth concentrations.

The results of Rg showed in Figure S2 that the fluctuation of Rg became larger and unstable at concentrations greater than 30%, suggesting that the stability of the matrix chain may deteriorate when ethanol (Eth) concentration is too high. In order to obtain the optimal concentration of Eth and to elucidate the molecular mechanism, the diffusion coefficient of the drug in the system and the radial distribution function (RDF) of SIN and matrix chain were analyzed in Figure 5. The results showed (Figure 5a) that the higher the content of Eth, the faster the diffusion coefficient of the drug and the more favorable for the release of the drug, due to the fact that the SIN was easily soluble in Eth; when the concentration of Eth was 30%, the degree of dissolution of the SIN was larger, and the distribution of the SIN in the gel matrix network was greater. The same conclusion was obtained by analyzing the RDF in Figure 5b, which indicates the probability of the appearance of SIN around the matrix chain and also indicates the strength of the polymer-SIN interaction. When the concentration of Eth is 30%, the probability of SIN around the matrix chain is the smallest, and the interaction is the weakest. This is conducive to the uniform distribution and release of the drug in the system. Therefore, the optimal concentration of Eth was screened as 30%.

### 2.6. Effect of Glycerine Concentration on the Quality of SIN Gel

**Figure 6:**
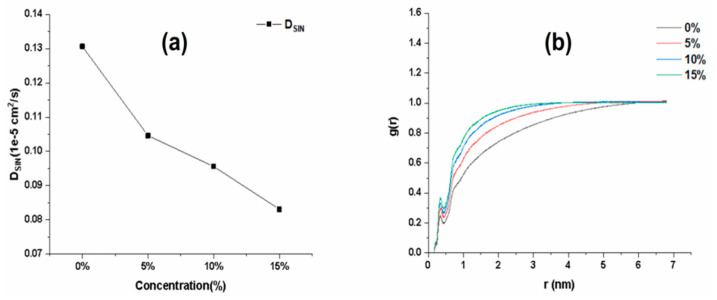
Effect of different Gly concentrations on the quality of SIN gel. (**a**) Diffusion coefficient for different Gly concentrations; (**b**) RDF values for different Gly concentrations.

The Rg values of matrix chains in the gel system with different glycerine (Gly) concentrations over time showed (Figure S3) that the Rg of the matrix chains became larger after the addition of Gly. This indicates that the greater the concentration of Gly, the slower the movement of matrix chain molecules and the more unfavorable the release of drug molecules. As shown in Figure 6a, SIN has the maximum diffusion rate without adding Gly. After adding Gly, the diffusion rate of drug release from the system decreases with the increase in Gly concentration. In real situations, Gly has a certain viscosity. The more Gly is added, the thicker the gelant becomes, which is more unfavorable for the release of drug molecules. The simulation results are in agreement with the theoretical results. To further explain this simulation result at the molecular level, this part also calculates the RDF of the oxygen atoms on the hydroxyl group of SIN with the hydrogen atoms in the water molecule. As shown in Figure 6b, the first sharp peak appears near 0.35 Å. As the concentration of Gly increases, the obtained peaks become higher and sharper, which indicates a strong interaction between the oxygen atoms and the hydrogen atoms. The RDF results also indicate the formation of a H-O-H type of hydrogen bonding. This result further proves that the addition of Gly affects the release of drug molecules in the matrix chain and the diffusion rate in the gel system. In conclusion, the concentration of Gly for this part of the screening was 5%.

### 2.7. Effect of Osmotic Promoters on Transdermal Penetration of SIN Gel

**Figure 7:**
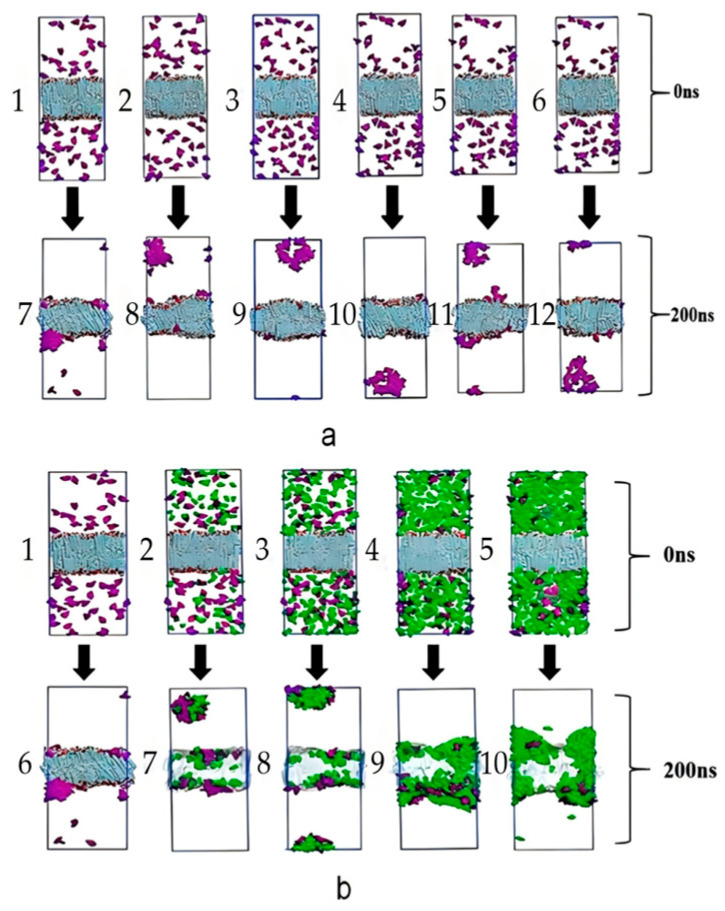
Effect of osmotic promoters on the quality of SIN gel. (**a**) Conformations of Proy and Lau systems with different concentrations at 0 ns and 200 ns (purple for SIN, blue for lipid bilayer); (**b**) conformations of Men systems with different concentrations at 0 ns and 200 ns (purple for SIN, green for Men).

When no pro-osmotic agent was added, the contact between SIN molecules and the skin was good, and SIN had a tendency to spontaneously infiltrate into the skin, indicating that SIN is lipophilic. There were fewer free molecules of SIN at a distance from both sides of the lipid bilayer. When 1,2-propanediol (Proy) and lauricnitrone (Lau) were added, although SIN was still in contact with the skin, the number of free molecules of SIN on both sides of the lipid bilayer increased. Moreover, SIN molecules were prone to agglomeration, which affected the spontaneous penetration of SIN into the lipid bilayer gap. As the concentration of menthol (Men) increased, the contact between SIN molecules and skin increased, and SIN and Men synergized to spontaneously infiltrate into the skin gap. In summary, Men can promote the transdermal absorption of lipophilic drugs. To further explain the molecular mechanism of the permeation promoter, the changes in the ordered parameters of ceramide-neurosphingosine (CER) and free fatty acids (FFA) in the skin bilayer model throughout the simulation were examined. As shown in Figure 7, the results indicated that the degree of change in the ordered parameters of CER and FFA in the lipid bilayer was greater with the addition of Men than with the addition of Proy and Lau alone or in combination. In order to further screen the optimal pro-osmotic agent and pro-osmotic agent concentration for SIN gels, therefore, the average diffusion rate of SIN from 0 to 200 ns was also calculated in this part for SIN at different pro-osmotic agent concentrations (Figures S4 and S5). The average diffusion rate of SIN from 0 to 200 ns was consistently greater with the addition of Men with the addition of Proy and Lau; the optimal pro-osmotic agent was determined in this section to be Men at a concentration of 10%.

### 2.8. Optical Microscopy

**Figure 8:**
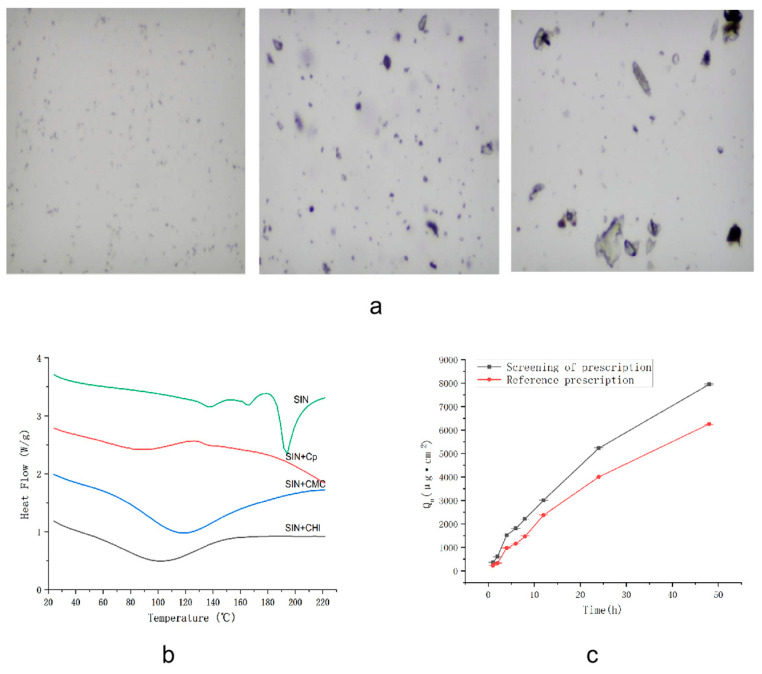
Traditional experimental validation. (**a**) Microscopic images at the same magnification (magnification: 4 × 10), in order of SIN and Cp matrix mixture, SIN and CMC mixture, and SIN and CHI mixture; (**b**) DSC thermogram; (**c**) in vitro transdermal penetration of the screening prescription and reference formulation.

The results of SIN and the mixtures of each matrix placed under the microscope are shown in Figure 8a. This indicates that SIN is more evenly distributed in the Cp matrix with fewer crystals, suggesting a greater degree of solubility. In contrast, in CMC and CHI, the distribution is uneven and the lumpy crystals are very large, indicating a low degree of solubility. The experimental results are in agreement with the simulation results.

### 2.9. Differential Scanning Calorimetry (DSC)

The DSC (Figure 8b) showed that the melting peak of SIN control product appeared around 150~165 °C [14]. The characteristic peaks of the physical mixture of SIN and Cp shifted to the left and the change in peak shape was small, indicating that the two can be compatible, which is consistent with the simulation results. The physical mixtures of SIN with CMC and CHI did not have obvious melting characteristic peaks, and the change in peak shape was large, indicating poor compatibility with CMC and CHI. The experimental conclusions are consistent with the simulation results.

### 2.10. In Vitro Transdermal Experiment

As shown in Figure 8c, the cumulative penetration amount of the SIN gel was greater than that of the reference preparation at 48 h, and the penetration rate of the SIN gel was greater. In summary, the cumulative penetration of the SIN gel screened by the MD method was superior to that of the reference formulation.

## 3. Discussion

In this paper, three matrix excipients (such as Cp, CHI and CMC) with low compatibility and low formulation risk with SIN were selected for formulation design. MD simulations were employed for δ calculations. Through this approach, Cp was identified as the optimal matrix. The screening results were validated using DSC and ordinary optical microscopy, and it was found that the simulation results were consistent with the experimental results, indicating that Cp was the optimal matrix for SIN gel. The interactions of the prescription factors in SIN gel were further investigated at the molecular level using all-atoms molecular dynamics (AA-MD) simulations. By calculating the Rg of the matrix chains of Cp and SIN, it was found that, the larger the Rg, the more unstable the matrix chains become. Moreover, the larger Rg leads to a slower diffusion rate in the gel. This condition is not conducive to releasing the drug molecules. At the same time, the density distribution is also a crucial factor influencing the release of SIN in the gel. The larger the density distribution of Cp polymer molecules, the quicker the release of the drug from the polymer network. RDF represents the probability of finding an atom (particle B) in a shelldr at the distancer of another atom (particle A) chosen as a reference point. By calculating the RDF between the oxygen atom and the hydrogen atom, as well as between drug molecules and polymers, it was found that a larger RDF value indicates a slower release of the drug. The principle of transdermal penetration of SIN gel lies in the fact that osmotic promoters modify the ordering parameters of the skin’s phospholipid bilayer, resulting in a change in the head-to-tail ordering of CER and FFA molecules in the skin. The enlarged gaps within the lipid bilayer, along with the disruption of its original order, made it easier for SIN drug molecules to enter the skin. Men disrupted the structure of the lipid bilayer, so SIN drug molecules could enter the skin and exert therapeutic effects in concert with the Men. This paper relied on an MD simulations approach to the examination of SIN gel at the molecular level; the optimal prescription was 3.0% of SIN, 1.0% of Cp, 30% of Eth, 5.0% of Gly, 10.0% of Men and pH of 6.5–7.0. The permeation rate and Qn of SIN gel screened by MD simulations methods were enhanced to a certain extent. This indicated that the methodology used in this paper is reliable and offered a relevant theoretical foundation and guidance for traditional experiments in the development and research of Chinese medicinal preparations. Based on SIN gel, this study aimed to conduct prescription screening and explore the molecular mechanism through MD simulation. It intended to elucidate the molecular mechanism principles that affect the quality of SIN gels and evaluate the quality of SIN gels. With the goal of promoting the rapid development of compliant gels, this study provided new ideas and a theoretical basis for the application of MD simulation in the field of pharmacy.

## 4. Materials and Methods

### 4.1. Materials

SIN was acquired from Chengdu Chenye Biot-echnology Co., Ltd. (Chengdu, China). Carbopol (Cp), chitosan (CHI), carboxymethylcellulose (CMC), lauricnitrone (Lau), ethanol (Eth) and menthol (Men) were obtained from Shanghai McLean Biochemical Science and Technology Co., Ltd. (Shanghai, China). Glycerol (Gly) and 1,2-propanediol (Proy) were supplied by Tianjin Guangfu Fine Chemical Research Institute (Tianjin, China). All other reagents and solvents used were of analytical grade.

### 4.2. Methods

#### 4.2.1. Calculating Solubility Parameter to Screen Matrix

The compatibility of a drug with a polymer matrix is usually evaluated using the solubility parameter (δ) [15,16]. δ describes the intermolecular interactions of a pure substance and is defined as the square root of the intramolecular cohesive energy density (CED). The δ is calculated in Equation (1).

(1) $$\delta =\sqrt{\mathrm{CED}}=\sqrt{\frac{E_{\mathrm{coh}}}{V_{m}}}$$

where CED is the cohesive energy of the substance, ${\;E}_{\mathrm{coh}}$ is the enthalpy of evaporation and $V_{m}$ is the molecular volume of the component.

The δ was calculated using the Amorphous Cell and Forcite modules of the software, and the COMPASS force field was chosen [17]. Since the carriers of the gel were polymers, the complete reduction of their molecular structure was resource-intensive [18], so polymers with a degree of aggregation of 2 were chosen uniformly. Four single-molecule structure optimizations are required for the construction of the periodic system. The constructed simulated periodic cells were optimized four times according to the same method. The Forcite module was applied to calculate the δ; the NVT system was selected for the equilibrium structure with a time step of 0.25 fs, a total number of simulation steps of 16,000 and a total simulation time of 4 ps.

#### 4.2.2. Prescription Screening of SIN Gel

**Figure 9:**
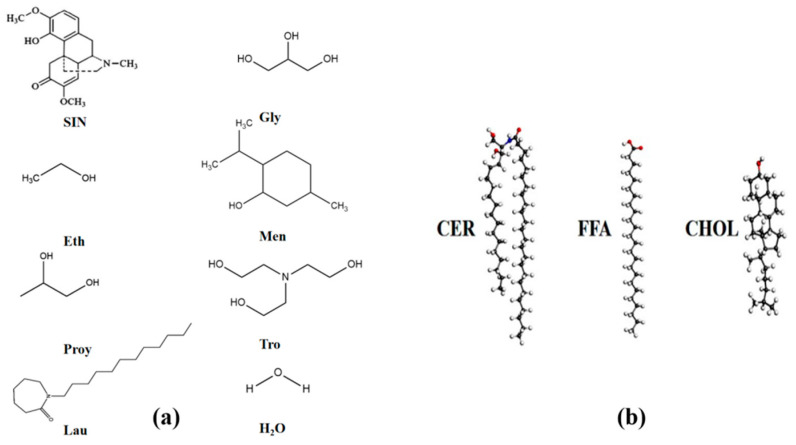
Molecular structure for MD simulations. (**a**) Molecular structural formulae of each prescriptive factor affecting the quality of the gel; (**b**) 3D structural diagrams of the molecules that make up the keratin bilayer of the skin: ceramide-neurosphingosine (CER), cholesterol (CHOL) and free fatty acids (FFA).

**Figure 10:**
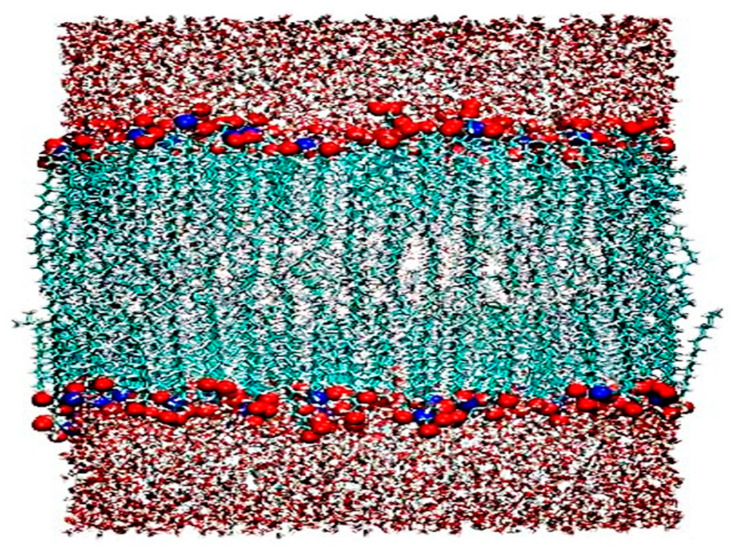
Skin bilayer model. The dark red area is aqueous solvent, and the head group oxygen and nitrogen of CER and FFA chains are shown in red, blue, and the main chains of CER, FFA and CHOL are uniformly shown in light blue.

In reference to the reference prescription (SIN 3%), (Cp 2%), (Eth 30%), (Gly 4%), (Proy 10%) and (Lau 2%), this study examined the effect of different concentrations of SIN, Cp, Eth, Gly and osmotic promoters (Proy, Lau, Men) to elucidate the effect of each prescribing factor on the SIN gel so as to screen the optimal prescription. The simulation details are as follows: a Generation Amber ForceField (GAFF) force field was selected [19]. The molecular topology files were generated with the help of Sobtop. In addition, the polymer topology was obtained by using Sobtop to call OpenBabel to recognize the Merck Molecular Force Field 94 (MMFF94) atomic charge [20]. The molecular structures of the study subjects and the construction of the skin keratin bilayer model are shown in Figure 9 for the molecular 3D structures of CER, cholesterol (CHOL) and FFA. To construct the skin lipid bilayer model, the structure was first adapted (http://samson-connect.net, accessed on 6 March 2022), and then the membrane-bilayer model structure was generated (http://sobereva.com/245, accessed on 6 March 2022), and, finally, applying GROMACS commands in conjunction with VMD 1.9.3 software, the constructed skin bilayer model is shown in Figure 10 [21,22]. MD simulations were carried out based on the GROMACS 2022.2 software package. Three-dimensional periodic boundary conditions were applied to all simulation regimes, and the steep energy minimization of 5000 steps was first executed with a time step of 1 fs, and the maximum force did not exceed 1000 KJ/mol. A pre-equilibrium simulation was performed in the NPT system for 10 ns, and the temperature was ramped up from 0 K to 298.15 K in 100 ps and then maintained at 298.15 K [23]. The temperature control method is V-rescale. The pressure is one standard atmosphere, and the pressure control method is Parrinello–Rahman. The Newtonian equations of motion are integrated using the velocity Verlet algorithm with a time step of 2 fs. The long-range electrostatic interactions are estimated using the PME method. The L-J pairwise interactions are evaluated within a cutoff distance of 10.0 Å [24]. The completed simulation is executed in the same way as the pre-equilibrium stage, with a simulation temperature of 298.15 K and a simulation time of 200 ns.

In order to evaluate the practical significance of the simulation results, the simulation system was inspected according to the following aspects: Total energy is used to evaluate whether the system has reached equilibrium, thus reflecting the reasonableness of the simulation time. RMSD is the most commonly used index to evaluate the degree of change in the solute structure relative to the initial structure during the MD process. The amplitude of the fluctuation is used to reflect the degree of stabilization of the polymer molecular structure. Rg is one of the important parameters to characterize the conformation of polymer molecular chains, and it is often used to determine the size of the polymer after swelling in water as well as its stretching ability in water. The larger the Rg, the greater the stretching ability. Density profiles refer to the distribution of particles in each cross-section perpendicular to a specific direction, reflecting the distribution of specific particles along a certain direction in the system. (In this paper, we mainly determine the density distribution of the gel system in the direction of Z-axis.) Diffusion coefficient is the diffusion speed of molecules or particles in the system during the simulation process. It can be measured by calculating the average displacement of particles in a certain time. RDF represents the probability density of other particles appearing at a certain distance from the reference particle. It is used to characterize the strength of the interaction force between molecules or atoms. Ordered parameters are used to describe the degree of ordering of molecules or atoms in the system. For bilayer structures, ordered parameters are often used to describe the degree of ordering or disruption of the molecular arrangement in the bilayer model.

#### 4.2.3. Optical Microscope Experiment

The drug was mixed with the dissolved matrix and observed under a microscope to understand the present state of the drug in the matrix, as well as its solubility and uniformity of distribution, so as to further validate the simulation results. First, 1.0 g of each matrix was weighed, 50 mL of deionized water was added, and it was mixed well and left for 24 h to make it fully dissolved. The dissolved gel matrix was stirred using magnetic stirring for 30 min (600 r/min) to produce a blank gel matrix. Then, 5.0 g of the dissolved blank matrix was weighed, 3 mg of SIN per portion was added, and the stirring time was controlled to be the same, so that the mixture was homogeneous, and the samples were prepared and set aside. The samples were uniformly applied to slides for microscopic observation with a microscopic magnification of 4 × 10.

#### 4.2.4. DSC

In order to verify the accuracy of the simulation results, the DSC method, a common method for evaluating the compatibility of drugs and excipients, was used to perform the compatibility test of drugs and excipient candidates. The test conditions were as follows: the heating rate was 10 °C/min, the temperature range was 25 °C~250 °C, and the control and physical mixed samples were measured [25].

#### 4.2.5. In Vitro Transdermal Experiment

By designing an in vitro transdermal assay, the in vitro skin permeation properties of SIN gel and the reference formulation were compared. The abdominal skin of rats was employed as an in vitro transdermal model [26]. The administration site is the center of the abdomen. The administration area is 1.5 cm × 1.5 cm. The skin was examined over a period of 48 h after application of SIN gel and the reference formulation to the skin. A Franz diffusion cell was used for the drug transdermal study, with the upper compartment serving as the drug supply cell and the lower compartment as the receiving cell. The experimental manipulation was carried out in a constant temperature water bath at 37 °C at 300 r/min. At the time points of 1, 2, 4, 6, 8, 12, 24 and 48 h, 1 mL of the receiving liquid was drawn from the receiving chamber as a sample and replenished with the same temperature and volume of phosphate buffer of pH 6.8. The content was determined using high performance liquid chromatography (HPLC) to assess the in vitro transdermal properties of SIN. The cumulative permeation volume was calculated as shown in Equation (2).

(2) $$Q_{n}=\sum_{n\;=1}^{i}C_{n}*V/S\;$$

where $Q_{n}$ is the cumulative infiltration per unit area of time, V is the receiving cell volume, C_n_ is the measured concentration of the nth sample and S is the effective infiltration area.

## 5. Conclusions

The compatibility between drugs and carriers serves as a pivotal determinant in assessing the drug-loading capacity of drug delivery systems. In this study, the δ of drug and excipients were calculated to predict the compatibility of the drug with different excipients as an index for screening formulation excipients. MD simulations were used to analyze the molecular microdynamic process to investigate the effect of prescription variables on the quality of SIN gel, and the prescription was successfully screened. It is noteworthy that this study represents a groundbreaking achievement in applying molecular simulation technology to the design and optimization of pharmaceutical formulations. It intuitively reflects the processes and phenomena that are difficult to demonstrate by experimental measurements, realizes the screening and optimization of drug delivery systems, reduces repeated exploratory experiments, and accelerates the process of drug formulation research and development.

## Figures and Tables

**Table 1:** Simulated values of SIN with candidate matrix (n = 3).

Systems	δ (J/cm^3^)^1/2^	∆δ (J/cm^3^)^1/2^
SIN	19.957	-
Cp	27.828	7.871
CHI	28.309	8.351
CMC	28.509	8.552

**Table 2:** Variation of solubility parameters of Cp polymers with degree of aggregations (n = 3).

Degree of Aggregations (n)	δ (J/cm^3^)^1/2^	∆δ (J/cm^3^)^1/2^
2	28.143	8.186
10	26.168	6.211
20	24.998	5.041
30	23.622	3.665
40	23.149	3.192
50	23.566	3.609

## Data Availability

Data are contained within the article and Supplementary Materials.

## Supplementary Materials

The following supporting information can be downloaded at: https://www.mdpi.com/article/10.3390/ijms252312863/s1.
