# Supplementary material for: Prescription Design of Sinomenine Gel Based on Molecular Dynamics Simulations
Source: Int J Mol Sci. 2024 Nov 29;25(23):12863. doi: 10.3390/ijms252312863 (PMC11640992; doi:10.3390/ijms252312863)
Supplement: Supplementary file 1 [file ijms-25-12863-s001.zip › ijms-3328177-supplementary.pdf]

Supplementary information

Fig S1. Rg variation curves for different Cp concentrations. (a) 0.5%; (b)1%; (c) 1.5%; (d) 2%; (e) 2.5%.

Fig S2. Rg variation curves for different Eth concentrations. (a) 0%; (b)20%; (c) 30%; (d) 40%; (e) 80%.

Fig S3. Rg variation curves for different Gly concentrations. (a) 0%; (b)5%; (c) 10%; (d) 15%.

Fig S4. Density distribution plots of different prescription factors and lipid bilayers in the system along the z-axis of the box. (a) Simulation results for Proy and Laur; (b) Simulation results for Men.

Fig S5. Ordering parameters of different osmotic promoters. (a) CER and FFA head ordering parameters in the lipid structure of the Proy and Lau; (b) CER and FFA head ordering parameters in the lipid structure of the Men; (c) CER and FFA tail ordering parameters in the lipid structure of the Proy and Lau; (d) CER and FFA tail ordering parameters in the lipid structure of the Men.

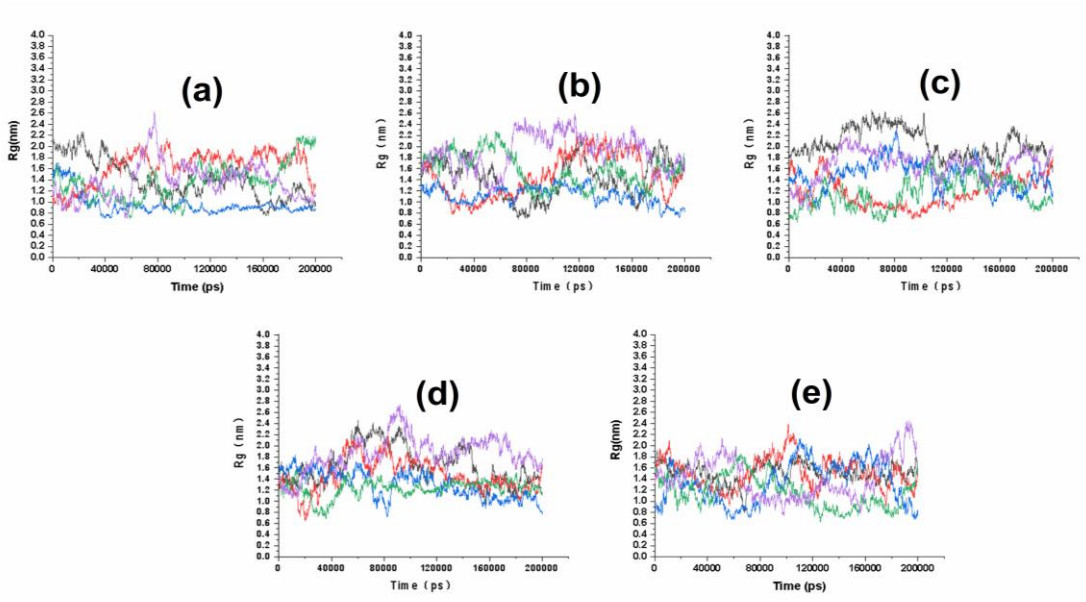

Fig S1. Rg variation curves for different Cp concentrations. (a) 0.5%; (b)1%; (c) 1.5%; (d) 2%; (e) 2.5%.

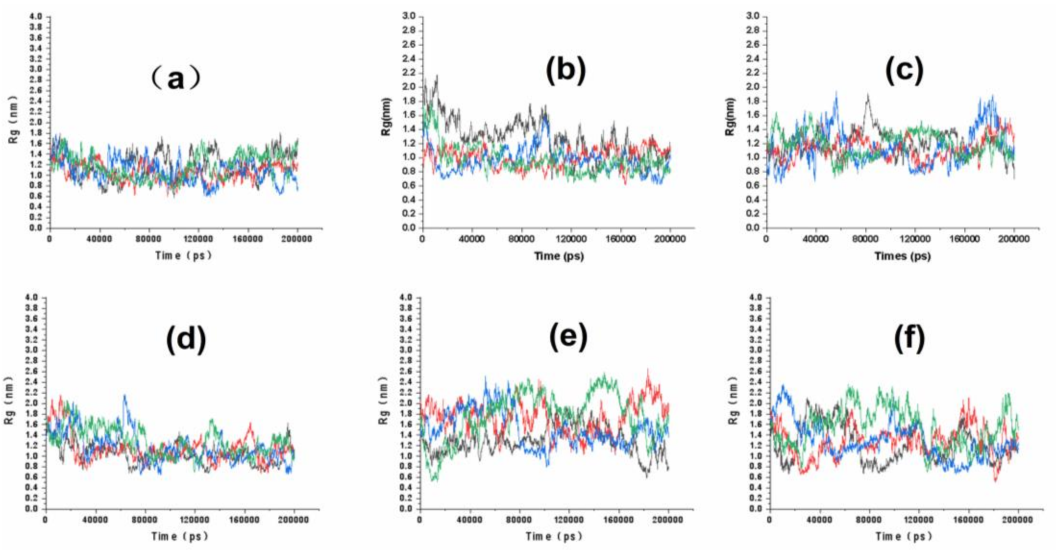

Fig S2. Rg variation curves for different Eth concentrations. (a) 0%; (b)20%; (c) 30%; (d) 40%; (e) 80%.

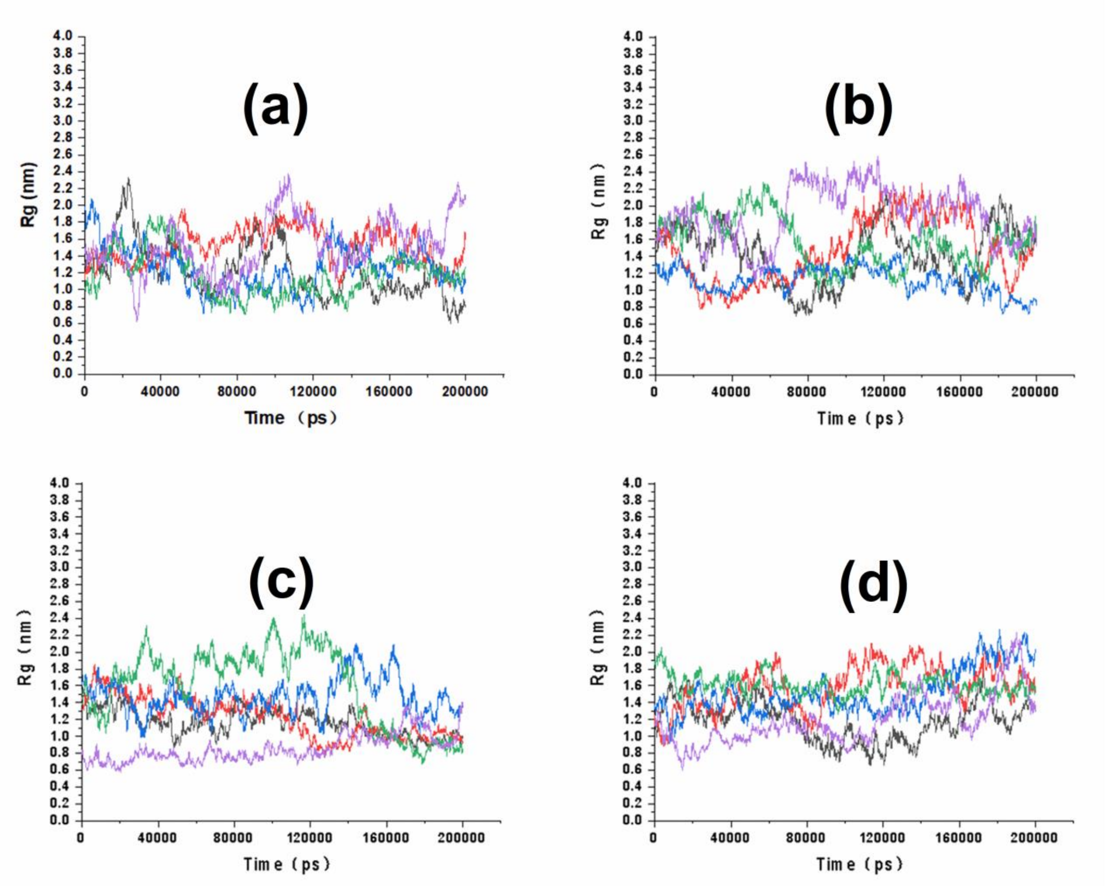

Fig S3. Rg variation curves for different Gly concentrations. (a) 0%; (b)5%; (c) 10%; (d) 15%.

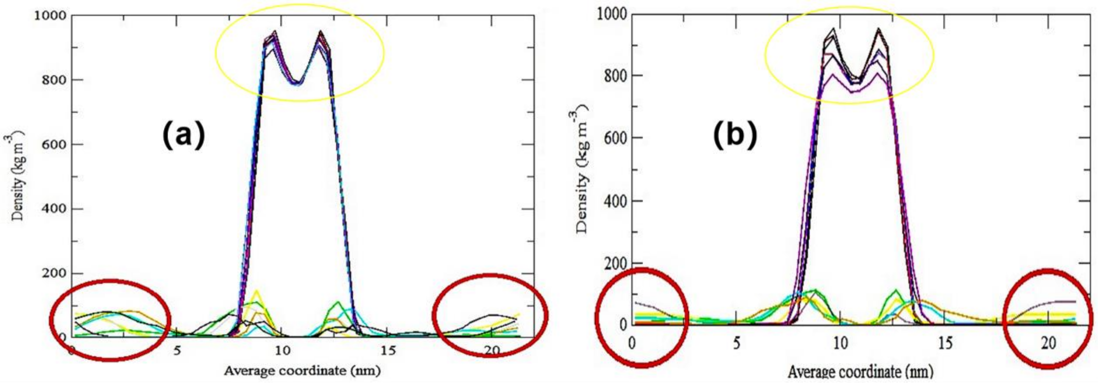

Fig S4. Density distribution plots of different prescription factors and lipid bilayers in the system along the z-axis of the box. (a) Simulation results for Proy and Laur; (b) Simulation results for Men.

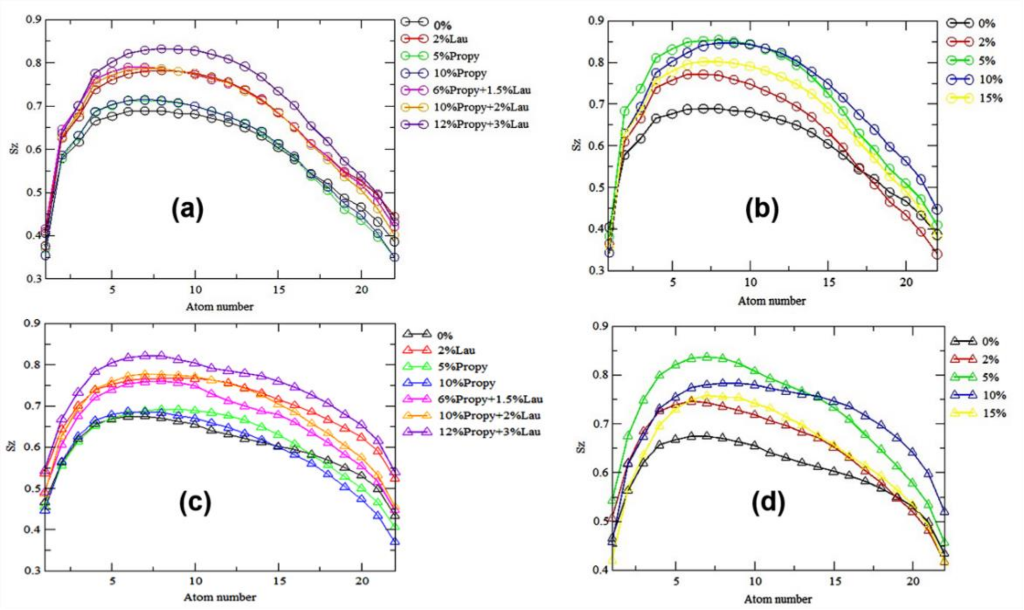

Fig S5. Ordering parameters of different osmotic promoters. (a) CER and FFA head ordering parameters in the lipid structure of the Proy and Lau; (b) CER and FFA head ordering parameters in the lipid structure of the Men; (c) CER and FFA tail ordering parameters in the lipid structure of the Proy and Lau; (d) CER and FFA tail ordering parameters in the lipid structure of the Men.
